# The CARF Protein MM_0565 Affects Transcription of the Casposon-Encoded *cas1-solo* Gene in *Methanosarcina mazei* Gö1

**DOI:** 10.3390/biom10081161

**Published:** 2020-08-07

**Authors:** Andrea Ulbricht, Lisa Nickel, Katrin Weidenbach, Herman Vargas Gebauer, Claudia Kießling, Konrad U. Förstner, Ruth A. Schmitz

**Affiliations:** 1 Institute of General Microbiology, Christian-Albrechts-University of Kiel, 24118 Kiel, Germany; aulbricht@ifam.uni-kiel.de (A.U.); lnickel@ifam.uni-kiel.de (L.N.); kweidenbach@ifam.uni-kiel.de (K.W.); hgebauer@ifam.uni-kiel.de (H.V.G.); ckiessling@ifam.uni-kiel.de (C.K.); 2 Core Unit Systems Medicine, Institute of Molecular Infection Biology, University of Würzburg, 97070 Würzburg, Germany; foerstner@zbmed.de; 3 ZB MED–Information Centre for Life Sciences, 50931 Cologne, Germany

**Keywords:** methanoarchaea, CRISPR-Cas system, transcriptional regulation, adaptation phase, casposon, *Methanosarcina mazei*

## Abstract

Clustered Regularly Interspaced Short Palindromic Repeat (CRISPR) loci are found in bacterial and archaeal genomes where they provide the molecular machinery for acquisition of immunity against foreign DNA. In addition to the *cas* genes fundamentally required for CRISPR activity, a second class of genes is associated with the CRISPR loci, of which many have no reported function in CRISPR-mediated immunity. Here, we characterize MM_0565 associated to the type I-B CRISPR-locus of *Methanosarcina mazei* Gö1. We show that purified MM_0565 composed of a CRISPR-Cas Associated Rossmann Fold (CARF) and a winged helix-turn-helix domain forms a dimer in solution; in vivo, the dimeric MM_0565 is strongly stabilized under high salt stress. While direct effects on CRISPR-Cas transcription were not detected by genetic approaches, specific binding of MM_0565 to the leader region of both CRISPR-Cas systems was observed by microscale thermophoresis and electromobility shift assays. Moreover, overexpression of MM_0565 strongly induced transcription of the *cas1-solo* gene located in the recently reported casposon, the gene product of which shows high similarity to classical Cas1 proteins. Based on our findings, and taking the absence of the expressed CRISPR locus-encoded Cas1 protein into account, we hypothesize that MM_0565 might modulate the activity of the CRISPR systems on different levels.

## 1. Introduction

In recent years, the CRISPR-Cas (clustered regulatory interspaced short palindromic repeat and CRISPR-associated) system has gained much interest due to its versatile usability for editing of target genomes (reviewed in [1,2,3]). Originally, the CRISPR-Cas system has been identified as a prokaryotic adaptive defense system against foreign nucleic acids like viruses, phages and plasmids and can be found in about one third of bacterial and nearly all archaeal genomes sequenced today [4,5,6,7,8]. A CRISPR locus is composed of CRISPR-associated (*cas*) genes, often organized in an operon, the CRISPR array, with variable numbers of direct repeats interrupted by invasive nucleic acid-derived spacer sequences and a leader sequence in front of it. The CRISPR-Cas function can be divided into three different stages (reviewed in detail, e.g., in [9,10,11]). In the adaptation stage, new spacers get incorporated into the CRISPR array after the attack of foreign nucleic acids. The second stage is the processing stage, with transcription of the CRISPR array as a long precursor CRISPR RNA (pre-crRNA), which is subsequently cleaved by CRISPR endonucleases and further processed into functional small crRNAs. Those crRNAs represent key elements of the CRISPR immunity, since in combination with different Cas proteins, they form the interference complex responsible for the degradation of foreign target DNA or RNA in the interference stage. Since CRISPR-Cas systems function as defense systems against foreign nucleic acids, they are assumed to be highly active in the presence of invading elements. Accordingly, for different prokaryotes, constitutive transcription of crRNAs has been demonstrated [12,13,14,15,16,17,18,19], emphasizing their need of a fast response to a huge variety of different invading elements. However, it is also known that some systems are induced, e.g., upon phage challenge, resulting in increased transcription of the CRISPR array and the associated *cas* genes, as it is reported for *Streptococcus thermophilus* [20], or general stress conditions. Stimulation of CRISPR-Cas transcription by foreign DNA was further substantiated in infection experiments using *Thermus thermophilus*, where transcription of both crRNA and cas genes was stronger than in uninfected controls [21]. In bacteria, several transcription factors either activating or repressing transcriptional activity within the respective CRISPR loci have been identified (reviewed in [18]), whereas the situation in archaea is much less clear. In early studies of different CRISPR-Cas systems, 135 potential regulatory genes were identified in close proximity to the respective CRISPR loci based on the analysis of 190 different archaeal CRISPR-Cas systems [22]. One of those, Csa3a from *Sulfolobus islandicus*, has been demonstrated to be of crucial relevance for the acquisition of novel spacers by activating the adaptation module of a type I-A CRISPR-Cas system [23]. Most interestingly, the transcriptional regulator Csa3b as well as the type I-A interference complex were also required for inhibition of transcription of the interference cassette [24].

The methanogenic euryarchaeon *Methanosarcina mazei* Gö1 contains two CRISPR-Cas systems, classified as subtype I-B and subtype III-C systems. Under standard growth conditions, both systems show very low activity with only a small amount of crRNAs detectable [25]. Moreover, challenging with Methanosarcina Sperical Virus (MetSV), the only virus so far known for *M. mazei*, always led to full infection and cell lysis. No spacer directed against this virus is present in the two CRISPR arrays of the *M. mazei* wild type [26], and until today, no spacer acquisition has been demonstrated due to virus challenge. Furthermore, analyzing CRISPR arrays from several *M. mazei* isolates from different habitats and locations revealed no MetSV-derived spacer with 100% sequence homology [26]. Whole genome proteomic analysis failed to detect the adaption proteins Cas1, Cas2 and Cas4, arguing for a strong repression of the adaptation module in *M. mazei* Gö1 under standard conditions [27]. Only in the presence of high salt concentration was an induction of the CRISPR-Cas systems in *M. mazei* detected with enhanced amounts of mature crRNAs from both CRISPR arrays [25].

Aiming to elucidate the proposed regulation of the CRISPR-Cas systems in *M. mazei*, we characterized the gene product of the open reading frame (ORF) *MM_0565*, which is located upstream of the type I-B system and was originally annotated as a transcriptional regulator and CRISPR-associated protein [4,28]. Using biochemical and genetic approaches, we evaluated its capability in *cas* gene regulation in *M. mazei* and obtained strong indications for its involvement in the CRISPR adaptation process.

## 2. Materials and Methods

### 2.1. Strains and Plasmids

Strains and plasmids used in this study are listed in Supplementary Table S1. Plasmid DNA was transformed into *M. mazei* as described by Ehlers et al. [29]. Plasmid DNA was, in general, transformed into *Escherichia coli* DH5α [30], JM109 λpir [31] or *E. coli* BL21-CodonPlus^®^-RIL (Stratagene, La Jolla, CA, USA), according to the method described by Inoue et al. [32].

### 2.2. Generation of Plasmids and Construction of Mutant Strains

For the MM_0565 overproducing mutant the gene, MM_0565 was PCR-amplified with the primers MM_0565_NdhI_for and MM_0565_BamHI_rev and TA-cloned into pCR4-TOPO (Invitrogen, Darmstadt, Germany) resulting in the plasmid pRS924. The plasmid pRS923 was created by restricting pRS924 by NdhI and BamHI and cloning of the respective 0.6 kilobase pairs (kbp) fragments into pET28a (+) (Novagene^®^, Merck Millipore, Darmstadt, Germany), restricted with NdhI and BamHI. Plasmid pRS1031 with the His_6_-tagged MM_0565 under control of the constitutive promoter pmcrB [33] was constructed as follows: pRS923 was restricted with NcoI and BamHI and the respective 0.65 kbp fragment was cloned into pRS893 (restricted with NcoI and BamHI), resulting in pRS1031. The plasmid pRS893 contains the constitutive promotor pmcrB [33] from *M. mazei* and its corresponding 5′ untranslated region (UTR) with a ribosomal binding site (RBS). For expression in *M. mazei*, the 0.7 kbp fragment was cloned with the help of SacI and KpnI into the pWM321 shuttle vector [34], resulting in pRS1032. pRS1032 was introduced in M. mazei* by liposome-mediated transformation as described by Ehlers et al. [29]. Transformants were selected based on puromycin resistance as colonies that grew on minimal medium plates with trimethylamine as the carbon and energy source, plus 5 µg puromycin/mL during incubation. Transformation was verified by detecting MM_0565 with a Western blot analysis [35] using a polyclonal antibody generated against MM_0565.

### 2.3. Growth of M. mazei

*M. mazei* strain Gö 1 was grown under anaerobic conditions at 37 °C with an atmosphere of 80% N_2_ plus 20% CO_2_ in 70 mL closed bottles in minimal medium containing 150 mM methanol as sole energy and C-source, according to Ehlers et al. [29]. The medium was supplemented with 100 μg/mL ampicillin to prevent bacterial contamination. For high salt stress, additional 500 mM NaCl were supplemented to the minimal medium. Cultures were grown until cells reached a turbidity at 600 nm of T_600_ 0.3 (high salt) or 0.5 (standard growth conditions) and harvested via centrifugation at 2.455× *g* for 30 min at 4 °C.

### 2.4. RNA Extraction

Total RNA was generally isolated by phenol extraction as described before [36] but using the Isol-RNA Lysis Reagent (5′ PRIME GmbH, Hilden, Germany, Cat. No. 2302700) followed by DNase I treatment, as described earlier [25].

### 2.5. RACE (Rapid Amplification of cDNA Ends) Analysis

To determine the transcriptional start site (TSS) of MM_0565, a RACE analysis was performed using the FirstChoice RLM-RACE kit (Ambion, Darmstadt, Germany) according to the manufacturer’s instructions but without calf-intestinal alkaline phosphatase (CIP) treatment. The specific oligonucleotides used are listed in Supplementary Table S2.

### 2.6. Quantitative RT-PCR

Transcript levels of genes in different strains and growth conditions were determined by quantitative (q) reverse transcription (RT)-PCR as described before [37,38]. The assays were performed using a QuantiTect Probe RT-PCR Kit (Qiagen, Hilden, Germany) and the ViiA™ 7 Real-Time PCR System (Applied Biosystems, Darmstadt, Germany). All primers used in this study are listed in Supplementary Table S2. The fold change in transcript abundance for each gene of interest was determined by comparison with the Cycle threshold (Ct) of three control genes (MM_1215, MM_1621 and MM_2181) which were known to remain constant during different growth phases and growth conditions [38].

### 2.7. Construction of cDNA Libraries for RNA-Seq and Illumina Sequencing

To construct RNA-seq libraries, two biological replicates of isolated total RNA from cells grown under standard growth conditions were used. cDNA library preparation was performed as described previously [39,40] with additional fragmentation followed by rRNA depletion using the Ribo-Zero rRNA removal Kit (Illumina, San Diego, CA, USA). For Illumina sequencing of cDNA molecules, the libraries were constructed by vertis Biotechnology AG (Freising, Germany) as described by Prasse et al. [40]. The cDNA libraries were sequenced using a HiSeq 2500 machine (Illumina, San Diego, CA, USA) in single-read mode.

### 2.8. Bioinformatical Analysis of the RNA-Seq Data

In order to assure a high sequence quality, the Illumina reads in FASTQ format were trimmed with a cut-off phred score of 20 by the program fastq_quality_trimmer from FASTX toolkit version 0.0.13 (http://hannonlab.cshl.edu/fastx_toolkit/). The following steps were performed using the subcommands “create”, “align” and “coverage” of the tool READemption [41] version 0.3.5 with default parameters. The poly(A)-tail sequences (introduced in the library construction) were removed and a size filtering step was applied in which sequences shorter than 12 nucleotides (nt) were eliminated. The collections of remaining reads were mapped to the reference genome sequences (NC_003901.1) using segemehl version 0.2.0 [42]. Coverage plots in wiggle format representing the number of aligned reads per nucleotide were generated based on the aligned reads. To restore the original data range and prevent rounding of small error to zero by genome browsers, each graph was then multiplied by the minimum number of mapped reads calculated over all libraries. Gene-wise read quantification was performed with READemption subcommand “gene_quanti”. Differential gene expression analysis was performed on these, counting with DESeq2 version 1.6.3 [43].

The RNA-seq data have been deposited in NCBI’s Gene Expression Omnibus [44] and are accessible through GEO Series accession number GSE151372 (https://www.ncbi.nlm.nih.gov/geo/query/acc.cgi?acc=GSE151372).

### 2.9. Purification of Heterologously Expressed His_6_-MM_0565

The N-terminally His_6_-tagged MM_0565 protein was expressed from plasmid pRS923 in *E. coli* BL21-CodonPlus^®^-RIL (Stratagene, La Jolla, CA, USA) growing in autoinduction media ZYP-5052, as described by Studier [44], in the presence of 30 µg/mL kanamycin. Cells were cultivated for a minimum of 24 h at 18 °C and vigorous shaking (120 rpm). Cells heterologously expressing MM_0565 protein for electrophoretic mobility shift assays were grown in lysogeny broth (LB)-medium in the presence of 30 µg/mL kanamycin and protein expression was induced at a T_600_ of 0.6 with 50 µM isopropyl-β-d-thiogalactopyranoside for 16 h at 18 °C. Cells were harvested at 4 °C and resuspended in His-tag buffer (250 mM NaH_2_PO_4_, 1.5 M NaCl, pH 8). After DNaseI was added, cell disruption was performed using a French pressure cell at 800 psi. The His-tagged fusion protein was purified from the respective cell-free extract by affinity chromatography using Nickel-Nitrilotriacetic acid (NTA) agarose (Qiagen, Hilden, Germany), as recommended by the manufacturer. After a washing step with at least 15 mL His-tag buffer supplemented with 75 mM imidazole, the MM_0565 protein was eluted in the presence of 150 mM imidazole. Protein used for electrophoretic mobility shift assays was eluted in the presence of 100, 250 and 500 mM imidazole. Finally, samples were diluted in His-tag buffer. The wash and elution fractions were analyzed by sodium dodecyl sulfate–polyacrylamide gel electrophoresis (SDS-PAGE) for quality control. If necessary, buffer exchange took place with the help of Amicon Ultra 0.5 mL centrifugal filters 10 K (Merck, Darmstadt, Germany), as recommended by the manufacturer.

### 2.10. Size-Exclusion Chromatography

The formation of the of His_6_-MM_0565 homodimers was determined by size-exclusion chromatography. Heterologously expressed and purified protein was separated in a mobile phase consisting of 50 mM N-[Tris(hydroxymethyl)methyl]-2-aminoethanesulfonic acid (TES) buffer pH 7.6 and 300 mM KCl using the ENrich™ SEC 650 column (BioRad Laboratories, Hercules, CA, USA) with a flow rate of 0.5 mL/min. Proteins were detected by monitoring the absorbance at 280 nm and 0.5 mL fractions were collected. Calibration of the column was performed using the gel-filtration mass standard (Bio-Rad Laboratories, Hercules, CA, USA) containing thyroglobulin (670 kDa), Immunoglobulin G (IgG) (158 kDa), ovalbumin (44 kDa), myoglobulin (17 kDa) and vitamin B12 (1.35 kDa).

### 2.11. Protein Analysis by Sodium Dodecyl Sulfate-Polyacrylamide Gel Electrophoresis (SDS-PAGE)

For separation and subsequent visualization of proteins, SDS gel electrophoresis was employed. For this purpose, protein containing solutions were supplemented with SDS-PAGE loading buffer (125 mM Tris-HCl pH 6.8, 140 mM SDS, 0.3 mM bromophenol blue, 10% β-mercaptoethanol (*v*/*v*)) and incubated at 95 °C for 5 min. Samples were analyzed by 12% SDS gel according to Laemmli [45]. To visualize protein bands, silver staining was performed. First, gels were washed with solution 1 (20 mL ethanol, 4 mL acetic acid, 16 mL H_2_O) overnight and after that, for 20 min with solution 2 (39 mL ethanol, 91 mL H_2_O) at room temperature. Silver staining was performed as described by Rabilloud et al. [46].

### 2.12. Protein Thermal Shift Assay

To test protein stabilization in different buffer systems at various pH values (pH 5.0 to 10.5) and salt concentrations (0 to 500 mM NaCl), a protein thermal shift assay was performed [47]. For this purpose, SYPRO™ orange dye (Thermo Fisher Scientific Inc., Waltham, MA, USA) was diluted 1:500 in various buffers containing 735 nM protein in a final volume of 20 µL. The assay was performed in the ViiA™ 7 Real-Time PCR System (Applied Biosystems, Darmstadt, Germany). The samples were heated from 25 to 95 °C by increasing temperature by 1 °C every minute. Changes in fluorescence were measured using the ROX filter set of the instrument. For calculating the melting temperature of the protein under various conditions, a Boltzman fit was performed.

### 2.13. Western Blot Analysis

Cell extracts (750 µg) or purified His_6_-MM_0565 protein were separated on 12% SDS-PAGE and transferred to nitrocellulose membranes (BioTrace^®^NT, Pall Life Sciences, Port Washington, NY, USA) [48]. Membranes were exposed to specific Penta-His™ antibody (Qiagen, Cat. No. 34660, Hilden, Germany) directed against the His-tag or with an antiserum generated against MM_0565. Protein bands were detected with secondary antibodies directed against mouse IgG or rabbit IgG coupled to horseradish peroxidase (Bio-Rad Laboratories, Hercules, CA, USA) and visualized using the ECL™ plus system (GE Healthcare, Chicago, IL, USA) with a fluoro-imager (DianaIII, Raytest, Straubenhardt, Germany).

### 2.14. Microscale Thermophoresis (MST)

For MST analysis, heterologously expressed His_6_-MM_0565 protein was purified as described above. 200 nM protein in Reaction buffer (His-tag buffer with 1 mg/mL bovine serum albumin (BSA) and 0.05% Tween20) was labeled with the Monolith His-Tag Labeling Kit RED-tris-NTA second Generation from NanoTemper (Munich, Germany) according to manufacturer instructions and followed by a 1:2 dilution in Reaction buffer and centrifugation for 10 min at 4 °C and 15,000× g. Double-stranded DNA (dsDNA) was produced by annealing two complementary oligonucleotides to a double strand. For this purpose, the two oligonucleotides were dissolved in annealing buffer (50 mM TES/KOH pH 7.6, 5 mM MgCl2 and 300 mM KCl) and mixed in equal amounts. After covering the solution with chill-out wax, the samples were boiled in water for 15 min and then allowed to acclimate to room temperature under gentle stirring. For measuring the concentration of the dsDNA, the Quant-iT™ dsDNA Assay Kit, broad range (Invitrogen, Carlsbad, CA, USA), was used according to the manufacturer’s instructions.

For all measurements, concentration of labeled protein was kept constant at 25 nM. A dilution series of nonlabelled dsDNA was prepared as described in the manual for the Monolith His-Tag Labeling Kit RED-tris-NTA (second Generation) Kit, with the exception that the dsDNA was diluted beforehand 1:2 with His-Tag Reaction buffer. The reaction mixture was incubated for 20 min on ice before measurement. All measurements were performed with the Monolith NT.115 (NanoTemper, Munich, Germany) at 25 °C using standard treated capillaries (NanoTemper, Munich, Germany), medium MST-Power and 100% Excitation-Power. K_D_-values were calculated for at least three biological replicates out of the normalized fluorescence change (ΔFNorm) using MO.Affinity Analysis software v2.2.4 (NanoTemper, Munich, Germany).

### 2.15. Electrophoretic Mobility Shift Assays (EMSA)

The leader regions of both CRISPR loci were amplified from *M. mazei* chromosomal DNA using the primer sets Leader_IB_for/KonsLeader_rev and Leader_IIIC_for/KonsLeader_rev. Radioactive labeling of DNA fragments and the following electrophoretic mobility shift assay was performed as described before [35] using the purified His_6_-MM_0565 protein (0.4–4 µM), dsDNA and binding puffer (50 mM Tris/HCl (pH 7.6), 750 mM KCl, 1 mM dithiothreitol, 0.5% Triton X-100, 62.5% glycerol (*v*/*v*) and 2.5 mM Ethylenediaminetetraacetic acid (EDTA)).

## 3. Results

### 3.1. MM_0565 Is a CRISPR-Cas-Associated Dimeric DNA-Binding Protein

**Figure 1:**
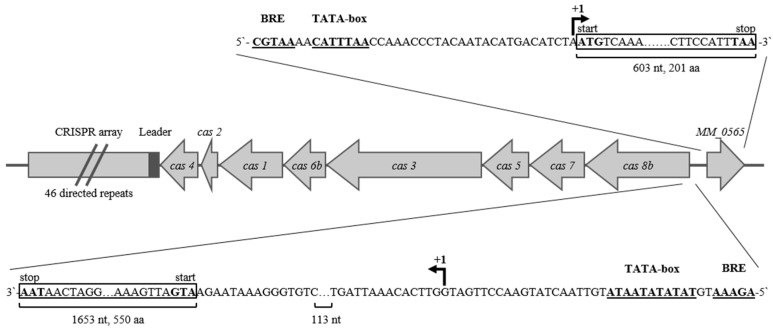
Localization and promoter region of the potential transcriptional regulator *MM_0565* and the Clustered Regularly Interspaced Short Palindromic Repeats (CRISPR)-CRISPR-associated (Cas) system I-B in *M. mazei* Gö1. The respective open reading frame (ORF) numbers are indicated according to Deppenmeier et al. [28]. Gene names are based on the nomenclature of Makarova et al. [4] and Verstergaard et al. [22]. B recognition element (BRE) and TATA box are indicated as well as transcriptional start sides (+1). Abbreviations: nucleotides, nt; amino acids, aa.

The ORF MM_0565 is located upstream of the type I-B CRISPR locus on the plus strand flanking *cas8b* (Figure 1). Using a differential RNA-seq analysis, the transcriptional start sites (TSS) of *MM_0565* and *cas8b* were determined. Accordingly, the respective promoters *MM_0565* and *cas8b* were identified (see Figure 1). In case of *MM_0565*, the TSS was further validated by a 5′ RACE analysis demonstrating that *MM_0565* transcript represents a so-called leaderless transcript. Both CRISPR-Cas systems of the subtype I-B (Figure 1) and the subtype III-C of *M. mazei* are located on the minus strand [25].

**Figure 2:**
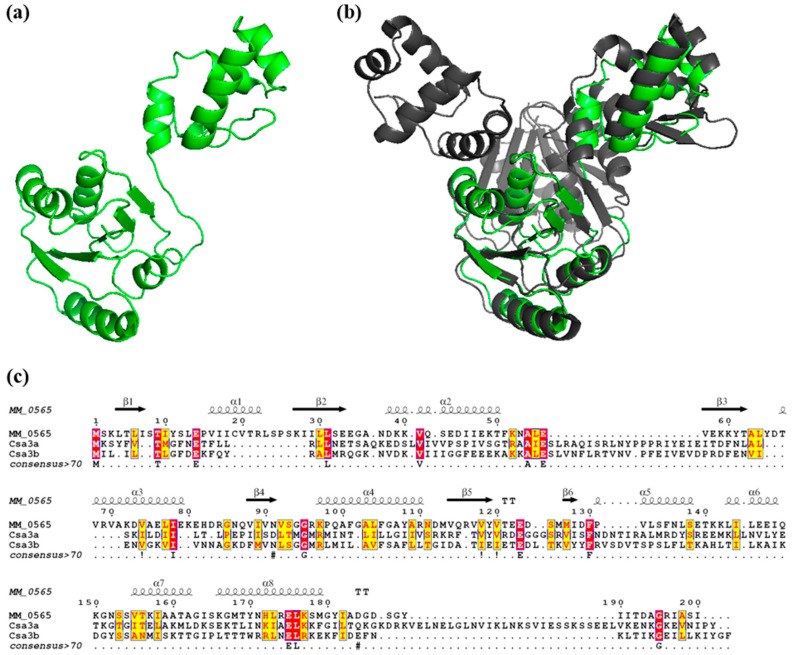
Structural model of the deoxyribonucleic acid (DNA)-binding protein MM_0565. (**a**) Structural model (Phyre2, Template: c2wteB, Confidence: 100, identity: 12%) of the DNA-binding protein MM_0565 of *M. mazei*. Depicted are the N-terminus with its Rossmann-like motif and the C-terminal winged helix-turn-helix domain. (**b**) Comparisons of the modeled structure of MM_0565 (green) with its nearest structural homolog the dimeric Csa3a (grey; PDB ID: 2WTE) from *S. solfataricus* [53]. (**c**) Structure-based alignment of MM_0565 with Cas3a and Cas3b from *S. solfataricus* P2 using Expresso [55]. The red boxes mark residues which are strictly conserved for all 3 sequences and the yellow boxes show amino acids which are conserved in 2 of the 3 sequences.

The predicted protein structure of MM_0565 was modeled using the Phyre2 web portal [49], revealing a two-domain structure of MM_0565 with an N-terminal CRISPR-associated Rossmann fold (CARF) and a C-terminal winged helix-turn-helix (HTH) domain (Figure 2a), as already reported by Makarova et al. [50]. While Rossmann-like folds typically indicate the potential to bind nucleotide co-factors, winged helix-turn-helix motifs are classically found in DNA-binding proteins that predominantly act as transcription regulators [51,52]. The nearest structural homolog of MM_0565 is Csa3a from *Sulfolobus solfataricus* (PDB ID: 2WTE), of which the crystal structure has been solved [53]. *S. solfataricus* Csa3a is a homo-dimeric protein which shows a very similar general architecture as *M. mazei* MM_0565 based on the two-domain structure described above. Although both proteins only share 12% amino acid identity (31% amino acid similarity), MM_0565 overlays perfectly with a monomer of the Csa3a-dimer from *S. solfataricus* (Figure 2b,c), with a Z-score of 21.0 calculated with the DaliLite server [54].

**Figure 3:**
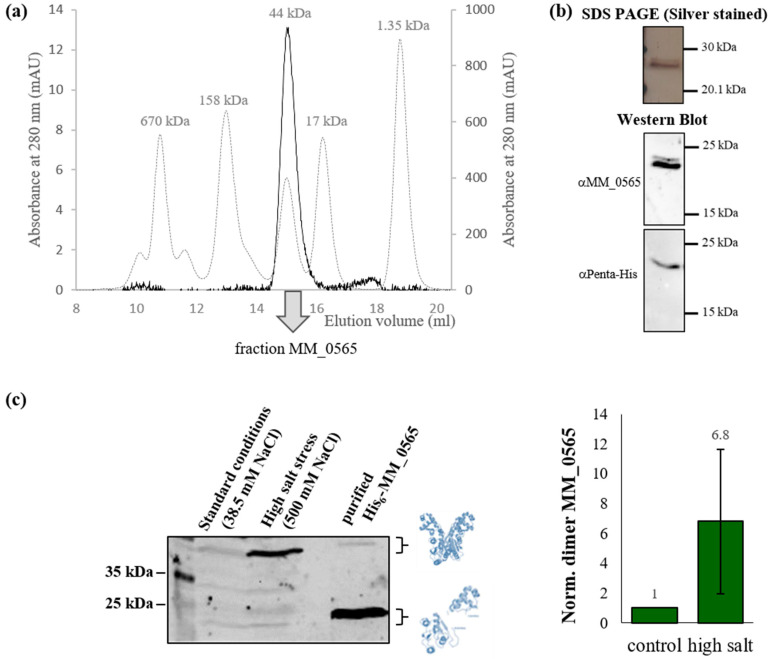
MM_0565 forms homodimers in solution. (**a**) Oligomerization of purified His_6_-MM_0565 was analyzed by size exclusion chromatography. For this purpose, His_6_-MM_0565 was separated in a mobile phase consisting of 50 mM TES buffer pH 7.6 and 300 mM KCl using an ENrich™ SEC 650 Column (BioRad Laboratories, Hercules, CA, USA), as described in Section 2. Black solid line: His_6_-MM_0565 (absorbance left y-axis), grey dotted line: Gel Filtration Standard from BioRad Laboratories (Hercules, CA, USA; absorbance right y-axis). (**b**) The fraction containing the main peak was analyzed by SDS PAGE analysis. The protein was detected by silver staining and immuno-blotting using antibodies raised against MM_0565 (αMM_0565) or the His-tag (αPenta-His). (**c**) MM_0565 protein levels of *M. mazei* cultivated under standard culture conditions (38.5 mM NaCl) or in media supplemented with 500 mM NaCl were analyzed by Western blot analysis using polyclonal antibodies directed against MM_0565 (depicted is one of four biological replicates). Band intensities were quantified densitometrically and normalized to the MM_0565 (dimer) levels occurring under standard growth conditions.

The biochemical properties of the MM_0565 gene product were determined by characterizing the heterologously expressed MM_0565 fused to an N-terminal His-tag, which purified to nearly 100% homogeneity by Ni-NTA affinity chromatography (approximately 22 kDa, Supplementary Figure S1). His_6_-MM_0565 showed high stability in TES buffer (pH 7.5) and an increased protein stability in the presence of sodium chloride concentrations up to 500 mM (highest concentration tested, Supplementary Figure S2). The oligomeric state of MM_0565 was analyzed by size exclusion chromatography on an ENrich™ SEC 650 Column. A single major peak was obtained for MM_0565 corresponding to a protein with a molecular mass of 44 kDa demonstrating that MM_0565 is a homodimer in solution (Figure 3a). The presence of MM_0565 in the respective fractions was verified by SDS PAGE and Western blot analysis using a commercial Penta-His antibody (Qiagen, Hilden, Germany) and polyclonal antibodies generated against MM_0565 (see Figure 3b).

The in vivo level of MM_0565 was analyzed by Western Blot analysis using polyclonal antibodies against MM_0565 under standard growth conditions as well as in the presence of 500 mM sodium chloride to evaluate the effect of high salt on its stability. In cell extracts from *M. mazei* grown under standard conditions, two protein bands with low intensity (25 and 50 kDa) were detected which correspond to the monomeric and the dimeric state of MM_0565 (Figure 3c, left panel). In contrast, the amount of dimeric MM_0565 in cells cultivated under high salt conditions (500 mM NaCl) was about 6.8-fold higher compared to standard conditions, which was accompanied by a general increase in MM_0565 protein levels. Importantly, the MM_0565 homodimer was detected despite a 5 min boiling step of the samples at 95 °C prior to the SDS PAGE under denaturing conditions (2% SDS), strongly arguing for a high affinity between MM_0565 monomers. This is an indication for a strong affinity to form dimers particularly, and induced in salt-stressed cells, verifying the results obtained in vitro (Figure 3a, in the presence of 300 mM KCl).

### 3.2. Regulation under Salt Stress and Binding of MM_0565 to the Leader Regions of Both CRISPR Arrays

**Figure 4:**
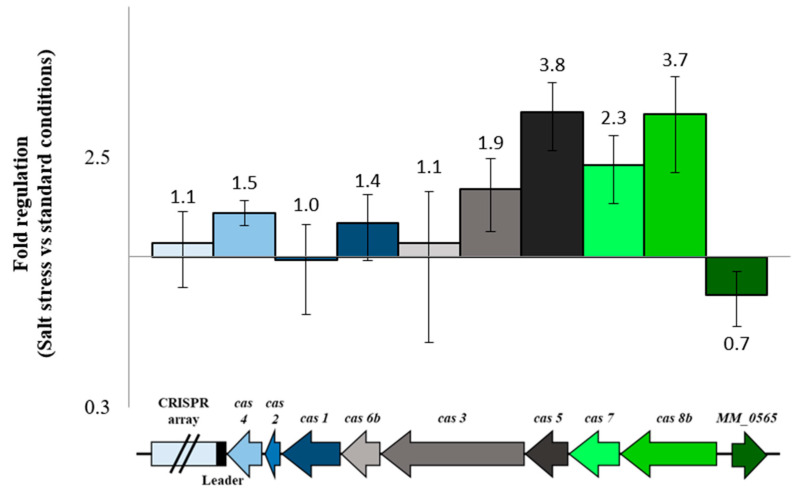
Regulation of subtype I-B CRISPR locus and MM_0565 under high salt conditions. *M. mazei* was cultivated under standard culture conditions (38.5 mM NaCl) or in media supplemented with 500 mM NaCl. mRNA levels of different CRISPR genes were analyzed by qRT-PCR in 3 biological replicates, as described in Section 2.

In the context of an induced amount of dimeric MM_0565 under high salt conditions, the transcription of the CRISPR-Cas I-B operon as well as the associated *MM_0565* gene was analyzed by quantitative (q) RT-PCR in salt-stressed cells in comparison to cells grown under standard conditions, since an induction of the CRISPR system in response to high salt was reported [25]. Under salt stress, the first four genes of the *M. mazei* CRISPR-Cas I-B operon (*cas8b*, *cas7*, *cas5* and *cas3*) were upregulated approximately two- to four-fold (Figure 4). The gene products of these genes encode proteins involved in the interference phase of CRISPR-based innate immunity. In contrast, genes, the product of which are responsible for the maturation of the crRNA (*cas6b*) or the adaptation of new spacers into the CRISPR array (*cas1*, *cas2* and *cas4*), were not significantly affected in their transcription (Figure 4). Interestingly, transcript levels of *MM_0565* were slightly but significantly downregulated by 30% under high salt conditions. This is in contrast to the strong increase in MM_0565 protein levels under these high salt conditions (Figure 3c), arguing for a significant stabilization of the protein, potentially by the formation of homodimers under high salt stress.

The binding potential of the putative transcription factor MM_0565 to the promotor of the CRISPR-Cas I-B operon (upstream of *cas8b*, see Figure 1) was analyzed. Evaluation of potential interaction of purified His_6_-MM_0565 to the *cas8b* promoter region using microscale thermophoresis (MST) clearly demonstrated that no specific binding of the purified protein was detectable (Supplementary Figure S3). Consequently, a direct effect of MM_0565 on the transcription of the *cas* I-B operon is very unlikely. Moreover, a direct protein–protein interaction of MM_0565 with the different Cas proteins was excluded by a pull-down experiment (using purified MM_0565 and *M. mazei* cell extracts) and subsequent mass spectrometric analysis of potential interaction partners.

**Figure 5:**
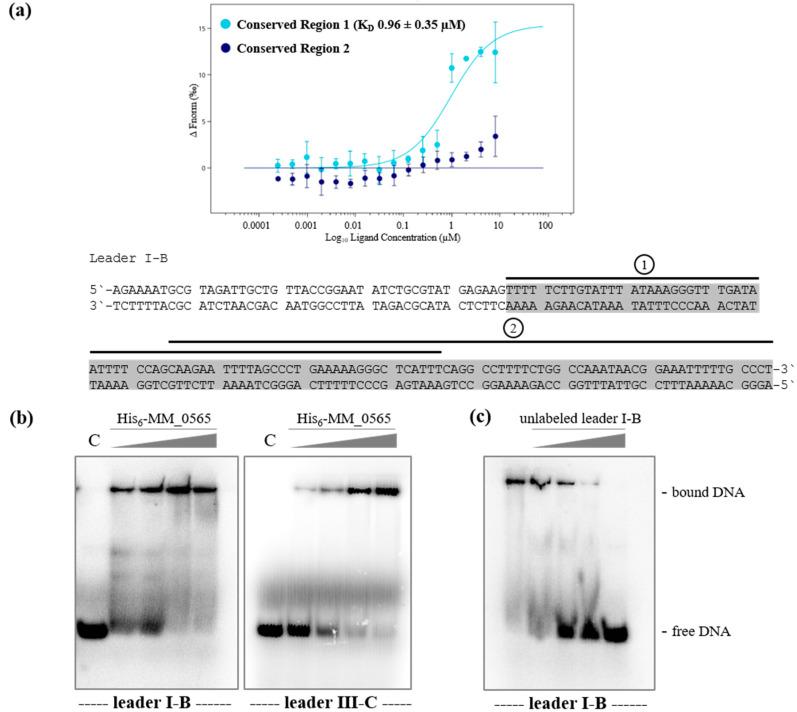
Binding of His_6_-MM_0565 to the conserved part of the leader regions in *M. mazei*. (**a**) Interaction between the fluorescently labeled protein His_6_-MM_0565 and two 70 nt long fragments of the conserved part of the leader from both CRISPR-Cas systems (5′ fragment, region 1; 3′ fragment, region 2). The interactions were evaluated and measured using the Monolith NT.115 from NanoTemper and *K*_D_-values were calculated of at least four independent replicates using the corresponding MO. Affinity Analysis Software (see Section 2). The grey box shows the conserved part of the leader of the two systems from *M. mazei*. The respectively tested dsDNA fragments are marked with a black line. (**b**) Binding assays with leader DNA from both CRISPR loci using decreasing concentrations of purified His_6_-MM_0565 (0.7–4 μM) and equal amounts of 5′-radioactively labeled leader DNA resulted in shift formation. The separation of samples was performed in native polyacrylamide gels (8%). Bands were visualized by phosphor-imaging. (**c**) Competition assay with 4 µM His_6_-MM_0565, constant amounts of 5′-radioactively labeled leader I-B DNA and increasing concentrations of unlabeled leader I-B DNA (8–120 ng), demonstrating specific binding of MM_0565.

Since the adaptation module in *M. mazei* seems to be repressed under standard growth conditions with no detectable Cas1, Cas2 and Cas4 proteins [27], and no MetSV-derived spacers were detectable after virus infection [26], we analyzed the ability of MM_0565 to bind to the leader region of the corresponding CRISPR-array, which contains important elements for the acquisition of new spacers into the CRISPR array [56,57,58,59]. The leader regions of the *M. mazei* type I-B and III-C CRISPR-Cas systems are highly conserved in their 3′ parts, with only three mismatches in the last 108 nt [25]. To test whether MM_0565 binds to the conserved leader sequence, MST was performed by using two different 70 nt long fragments of the conserved 108 nt leader part of the type I-B system (see Figure 5a). While binding to the 3′ fragment (position 38 to 108 of the conserved leader) was not detected (Figure 5a, region 2, dark blue dots), purified His_6_-MM_0565 efficiently bound the 5′ fragment (the first 70 nt of the conserved 108 nt leader part, Figure 5a, region 1, light blue dots) with a *K*_D_ of 0.96 ± 0.35 µM (see Figure 5a), which was confirmed by two independent biological replicates. To further verify this finding, electrophoretic mobility shift assays were performed with the purified His_6_-MM_0565 and 5′ radioactive-labeled leader DNA from both CRISPR loci (Figure 5b). The analysis showed that His_6_-MM_0565 was able to bind to either leader with an apparently higher binding affinity to the subtype I-B leader DNA. Specific binding was further validated for the subtype I-B leader by a competition assay titrating increasing amounts of unlabeled DNA into the assay (Figure 5c).

### 3.3. MM_0565 Globally Affects Transcription in M. mazei

In order to further analyze the function of MM_0565 in vivo, we performed a genetic approach. While an overexpression mutant was generated as described in Section 2, despite several attempts and using different approaches, generation of a MM_0565-deficient strain was not successful, arguing for an essential gene function in *M. mazei* Gö1. Constitutive overproduction of MM_0565 (*M. mazei* strain OP MM_0565) led to no significant effects on growth under standard conditions when compared to the vector control. Consequently, we analyzed the transcriptome of *M. mazei* with additional expression of MM_0565 employing an RNA-seq approach in comparison with the wild type containing the respective but empty vector. This analysis described in detail in Section 2 discovered 55 genes differentially transcribed due to MM_0565 overproduction. Of these genes, 15 showed a reduced and 40 an increased transcript level (Table 1). The majority of genes with an upregulated transcript level in the *M. mazei* OP MM_0565 strain encoded for key enzymes of the central carbon and energy metabolism. On the other hand, four energy metabolism-related genes were identified to be less abundant. Additionally, besides genes of unknown functions, genes coding for membrane-associated proteins and transposases were differentially transcribed upon MM_0565 overproduction (Supplementary Figure S4a).

To identify properties of potential binding sites of MM_0565, the promotor regions of the differentially transcribed genes identified in the RNA-seq data were aligned. For the promotor regions (from position −100 to −1) of a total of 14 significantly upregulated genes, a frequency blot was created using the Weblogo tool [60]. Putative TATA-boxes and B recognition elements (BRE) were identified (see Supplementary Figure S4b). Besides the TATA-boxes, the promotor regions showed an overall AT-rich sequence upstream of the promotor and directly downstream of the TATA box, but a distinct binding motif was not identified. These AT-rich elements were not detected in promotor regions of genes with a decreased transcript level. Based on those findings, we conclude that MM_0565 functions as a transcriptional activator in many cellular processes including essential ones, which might be the reason why a deletion mutation is not stable. Nevertheless, additional repressive functions of MM_0565 or indirect effects cannot be excluded at this point.

**Figure 6:**
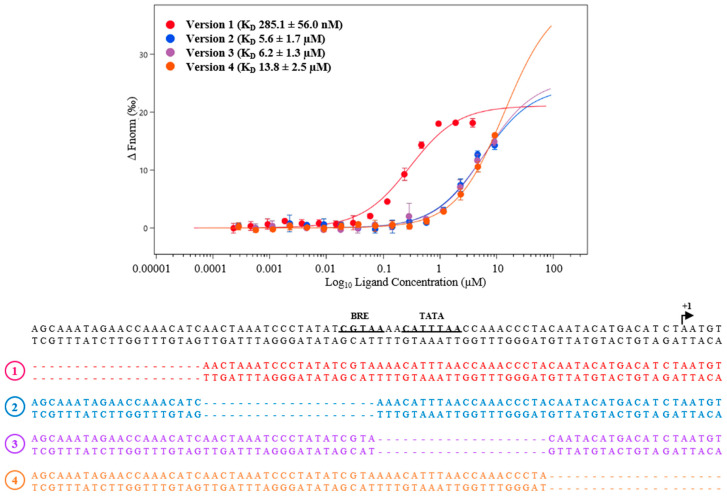
Specific binding of His_6_-MM_0565 to its promoter. Interaction between the fluorescently labeled protein His_6_-MM_0565 (25 nM) and the promoter regions of the gene MM_0565 (wild type (wt) (version 1) and derivatives (version 2–4)) was analyzed by microscale thermophoresis (MST). The diagram shows the binding curve of four different versions of the promoter region of MM_0565, each lacking different 20 nt fragments. All interactions were measured using the Monolith NT.115 from NanoTemper and *K*_D_-values were calculated of at least three independent biological replicates using the corresponding MO. Affinity Analysis Software (see Section 2).

Activity of transcription factors is often tightly controlled. Often, these factors also bind to their own promotors to inhibit their expression in a negative feedback loop. Thus, we studied potential autoregulation of MM_0565. MST analysis revealed binding of purified His_6_-MM_0565 to its own promotor with a calculated *K*_D_ of 285.1 ± 56.0 nM (Figure 6). To further narrow down the actual binding site of MM_0565 in this region, we tested several mutant derivatives of the respective DNA fragment. The 60 nt dsDNA promoter region was divided in 3 parts each consisting of 20 nt. Deletion of any of these three parts led to a strongly reduced binding affinity of MM_0565 to the corresponding promoter region fragments, which also encompass the transcription-relevant BRE-element and TATA-box (Figure 6). This finding strongly argues for the requirement of certain specific DNA-elements covering the whole analyzed DNA fragment.

**Figure 7:**
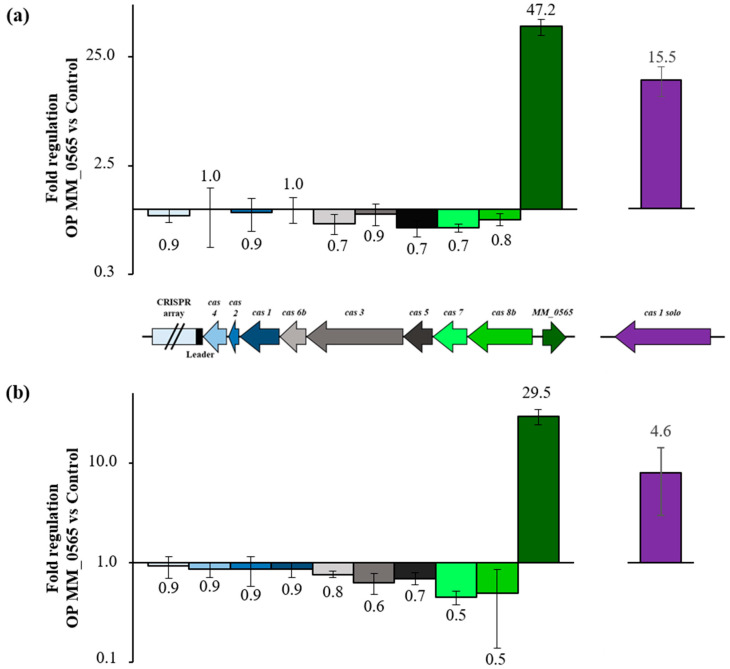
Effect of MM_0565 overproduction in *M. mazei*. Regulation of *M. mazei* type I-B CRISPR locus genes and *MM_0565* in the overproduction mutant of MM_0565 (**a**) under standard growth conditions and (**b**) under high salt stress (500 mM NaCl). (**b**) mRNA levels of different CRISPR genes were analyzed by qRT-PCR in three biological replicates (for details see Section 2).

Despite the close association of MM_0565 with the type I-B CRISPR locus of *M. mazei*, the employed RNA-seq experiment did not reveal any alteration in the expression of *cas* genes due to overproduction of MM_0565, which is in agreement with the low potential of MM_0565 to bind to the promoter of the CRISPR-Cas I-B operon. This was further verified by a qRT-PCR analysis of the corresponding genes comparing the overproduction mutant of MM_0565 with the empty vector control under standard growth conditions (Figure 7a). Even under osmotic stress (500 mM NaCl), the overproduction of MM_0565 in *M. mazei* showed no significant influence on the expression of the genes of the type I-B CRISPR locus (Figure 7b). This was also the case for *cas8b*, *cas7*, *cas5* and *cas3* which were upregulated under high salt concentrations in the wild type (Figure 4). In addition, no effect on the transcription of the crRNA was observed upon overproducing MM_0565, neither by quantitative RT-PCR nor by Northern blotting.

**Figure 8:**
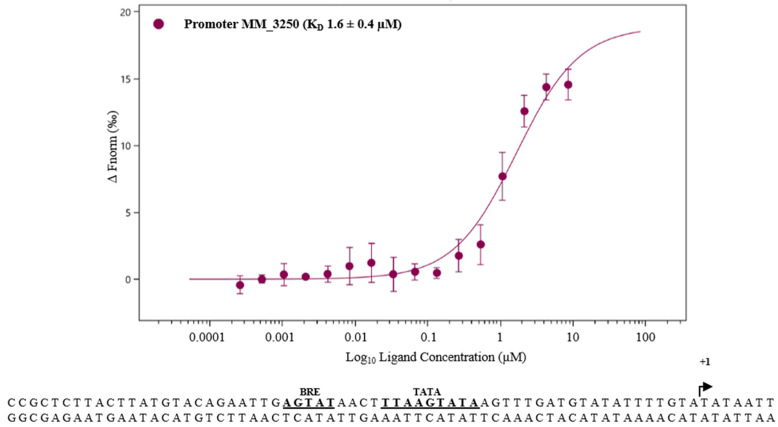
Interaction between His_6_-MM_0565 and the promoter of *MM_3250*, demonstrated by MST. Interaction between the fluorescently labeled protein His_6_-MM_0565 (25 nM) and the promoter region of the gene *MM_3250* was analyzed using the Monolith NT.115 from NanoTemper. The *K*_D_-values were calculated of at least three independent replicates using the corresponding MO.Affinity Analysis Software (see Section 2).

Consequently, no obvious link that MM_0565 is a transcriptional regulator of the CRISPR locus-encoded *cas* genes of *M. mazei* was obtained from the quantitative RNA-seq experiment. However, detailed analysis of those differentially transcribed genes which are not assigned to a function according to Deppenmeier et al. [28], using recent later genome annotations [61] and further protein information, demonstrated that one of the differentially regulated genes (ORF MM_3249) was identified as a novel *cas1* gene not associated to the CRISPR-Cas loci. *MM_3249* is encoded in an operon together with *MM_3250*, for which no function was assigned. Both genes showed a significantly increased transcript level due to MM_0565 overproduction (see Table 1). This standalone *cas1* gene, was recently designated *cas1-solo*, and based on its genomic location and gene environment, it was characterized as part of a mobile genetic element, the so-called casposon [62]. This Cas1-solo and the CRISPR locus I-B-encoded Cas1 (*MM_0559*) protein in *M. mazei* share an amino acid identity of 26% (amino acid similarity 44%). The effect of MM_0565 overproduction on *cas1-solo* transcript level was verified by qRT-PCR analysis in comparison with CRISPR locus I-B-encoded *cas1* (MM_0559). This demonstrated that due to overproduction of MM_0565, the mRNA level of *cas1-solo* was enhanced about 36-fold (Figure 7). In agreement with previous findings, no significant changes in the transcriptional level for *cas1* were determined. To verify the direct upregulation of *cas1-solo* by MM_0565, we examined the binding of purified His_6_-MM_0565 to the promoter region of the corresponding operon (*MM_3249*-*MM_3250*) by MST (Figure 8). Specific binding of MM_0565 to the promoter region of the operon including *cas1-solo* was demonstrated with a calculated affinity of 1.6 ± 0.4 µM. This finding clearly provided evidence that MM_0565 is able to activate the transcription of the *cas1-solo* gene, whose promotor region is also characterized by a high AT-density in addition to the TATA-box (Figure 8).

## 4. Discussion

Tight regulation of CRISPR-Cas modules is often pronounced in different model organisms. In *E. coli*, for example, the histone-like structuring protein (H-NS) negatively regulates *cas* gene expression by inhibiting the transcription of the *cas* operon due to binding near the promotor region [63,64], another example is the lysine-responsive regulatory protein (LRP), a negative regulator of the *cas* operon in *Salmonella typhi* [65]. Due to an overall low activity of the CRISPR-Cas system in *M. mazei* Gö1 and repression of the CRISPR locus type I-B-encoded adaption module [25,27], we proposed a tight control of this system, the elucidation of which was the focus of this report, analyzing the CRISPR-associated DNA-binding protein MM_0565 and evaluating its potential effects on CRISPR-Cas regulation in *M. mazei*.

### 4.1. MM_0565, Structural Features and Its Link to CRISPR-Cas Systems

Based on its structural features, MM_0565 can be grouped into the superfamily of CRISPR-Cas-associated Rossmann-fold (CARF) domain proteins [50]. These proteins often occur in close proximity to CRISPR loci. In addition to their CARF domain, they contain various effector domains including different nucleases and/or regulatory domains such as winged HTH (wHTH), which was also shown for MM_0565 (Figure 2, and Makarova et al. [50]), highlighting the potential of CARF proteins to function as central regulators of CRISPR-Cas systems [4,6,61].

The broad versatility of CARF proteins can be illustrated by the two CRISPR-associated CARF proteins Csa3a from *S. solfataricus* and Csa3b which was described for different *Sulfolobus* strains. On the one hand, Csa3a, the closest structural homolog of MM_0565, has been demonstrated to function as a transcriptional activator of the expression of type I-A adaptation genes while simultaneously, it increases the expression of crRNA and genes which are involved in DNA repair mechanisms [66]. On the other hand, Csa3b acts as a transcriptional repressor of *cas* genes which are involved in the formation of the interference complex [24]. These different functions in either activation or repression of CRISPR-Cas systems reflect what was verified for the CARF protein MM_0565 in *M. mazei* in this study.

### 4.2. MM_0565 Has No Direct Effect on Type I-B cas Gene Transcription

Using a genetic approach, we demonstrated that MM_0565 affects a broad range of different genes, the product of which fulfill various cellular functions ranging from energy metabolism to DNA modification and membrane-associated processes (Supplementary Figure S4 and Table 1). This highlights that MM_0565 most likely has a broad repertoire of cellular targets and is involved in pathways exceeding the adaptive immunity of *M. mazei*. In general, MM_0565 appears to prefer AT-rich DNA sequences as binding sites, as demonstrated by analysis of the promotor regions of the genes upregulated by this protein (Supplementary Figure S4b). However, no distinct binding motif has been identified so far and we cannot exclude that several identified genes might be indirectly regulated by MM_0565 by effecting expression of other transcriptional regulators or masking of a regulatory effect by MM_0565 due to an already overall repressed CRISPR system.

The prominent role of MM_0565 in *M. mazei* was further substantiated by the failure to generate a MM_0565-deficient strain despite employing various strategies. This clearly points to an essential function of this protein in *M. mazei*.

*M. mazei* only possesses a low activity of the CRISPR-Cas systems which is in line with a very low to absent expression of the Cas proteins under steady-state conditions [27]. Similarly for *E. coli* and *Salmonella enterica* serovar Typhi, it was shown that the heat-stable nucleoid-structuring protein (H-NS) in parallel to its function as a global repressor also regulates *cas* genes’ expression in both organisms, resulting in low activities of the respective systems [63,65]. However, in *M. mazei,* a transcription regulatory function of MM_0565 on any of the *cas* genes of the associated type I-B CRISPR-Cas system under any conditions tested was not detectable using different approaches (Figure 7). Furthermore, MM_0565 did not influence expression of crRNAs (in a *M. mazei* MM_0565 overproduction mutant), as demonstrated by Northern Blot analysis. Intriguingly, expression of several *cas* genes and maturation or stability of crRNA is significantly induced by osmotic stress (500 mM NaCl) [25]. Under those conditions, *MM_0565* transcript levels were mildly downregulated but the corresponding protein was significantly stabilized and predominantly detected as a robust dimer (Figure 3), which most likely represents the physiologically active form of the protein and emphasizes the auto-regulatory function of MM_0565 (Figure 6). Even though recombinant MM_0565 appears to be stabilized by increasing sodium chloride or other compatible solutes concentrations (Figure 3c and Supplementary Figure S2), the concrete mechanism(s) of potential MM_0565 contribution to the salt-dependent effect on crRNAs and *cas* genes in vivo so far remain enigmatic and might be explained by the liberation of a so far uncharacterized allosteric effector that is able to bind to the CARF domain of MM_0565 to modulate its regulatory function and dimerization potential. Alternatively, MM_0565 might bind to the promoter of the type I-B CRISPR-Cas operon employing a so far enigmatic/unknown and rate-limiting co-factor or interaction partner that itself is regulated by the presence of sodium chloride in the culture medium. The existence of a low-abundant but essential co-factor might also explain the low influence of overexpression of MM_0565 on the expression of *cas* genes under standard conditions. Thus, no direct effects of MM_0565 on the transcription of type I-B *cas* genes or the crRNA was obtained, but due to stability effects during high salt conditions on the MM_0565 dimer, and especially the crRNAs, a link between the two cannot be ruled out. Lastly, it should be taken into account that in case MM_0565 acts as a negative regulator of *cas* genes in *M. mazei*, the effect of an overexpression on an already repressed system might be hard to validate.

### 4.3. MM_0565 Might Influence Activity of CRISPR-Based Immunity by Binding to the Leader

MM_0565 directly interacts with the conserved region of the leader DNA of both CRISPR-Cas systems in *M. mazei* Gö1, which was proven by MST analysis (Figure 5a). The conserved sequences are approximately 100 nt long DNA elements downstream of the CRISPR array promotor regions [25]. In general, leader sequences typically contain sequence motifs important for binding of the Cas1-Cas2-complex and other cellular factors which are important for the adaptation of new spacers into the CRISPR array [58,67]. Thus, binding to the leader regions of the CRISPR arrays represents an interesting mode of regulation of CRISPR-Cas-mediated immunity by MM_0565 and it appears likely that MM_0565 directly affects recruitment of the Cas1/Cas2 complex or might also modulate structural features of the leader sequences that have an impact on the integration of new spacers into the CRISPR array. This was demonstrated for the integration host factor (IHF) from *E. coli*. In addition to its genome-wide regulatory functions, IHF is involved in the adaptation of new spacers by binding to a conserved motif 60 bp upstream of the first repeat within the leader sequence. It therefore determines the appropriate integration site for new spacers at the leader-repeat-junction in the I-E CRISPR-Cas system [68] and minimizes the costs for an efficient immune answer by facilitating the contact for the Cas1-Cas2 integrase anchoring site [69,70]. This post-transcriptional function of IHF in the CRISPR-Cas system from *E. coli* and its otherwise more global role is reminiscent of the diverse cellular functions of MM_0565, discovered in our genetic approach, and also its putative post-transcriptional involvements in the function of the CRISPR-Cas system from *M. mazei*.

### 4.4. MM_565 Highlights the Position of Casposons as Ancesterors of CRISPR-Cas-Encoded Cas1 Proteins

As discussed above, MM_0565 does not appear to directly influence transcription of *cas* genes but rather impacts on the CRISPR-Cas systems by binding to the leader of the two CRISPR arrays. Interestingly, with respect to the MM_0565 overproduction in *M. mazei*, the transcriptional activation of a standalone *cas1* (*cas1-solo*) gene was observed during this study (Table 1) and was verified by MST analysis (Figure 8). *Cas1-solo* genes are part of a new family of self-synthesizing large DNA-transposons, the so-called casposons, which appear to rely on Cas1-solo for integration and excision and were proposed to be an ancestor of CRISPR adaptation modules [62]. Casposon mobility was shown for different *M. mazei* strains [71], supporting the hypothesis of casposons as a new group of mobile genetic elements. Stress-induced mobility is known for transposable elements (reviewed in [72]) and might explain the connection between the enhanced stability of the dimeric MM_0565 and the activation of the casposon-encoded Cas1-solo protein. Furthermore, the mechanism of spacer integration mediated by Cas1 proteins is very similar to the transposition reaction proposed for casposons, which was further supported by structural analysis of the *M. mazei* Cas1-solo by Hickman et al. [73]. There, it was highlighted that casposon end-binding by Cas1-solo resembles that of spacer 3′ overhangs during the adaptation phase. Thus, an evolutionary link between casposons and the CRISPR-Cas system seems very likely. The active site of the CRISPR locus-encoded Cas1 protein from *E. coli* was studied via site-directed mutagenesis and the corresponding crystal structures revealed four conserved residues [74], which are all conserved in the *M. mazei* Cas1-solo [62] and as well in the CRISPR locus-encoded Cas1 in *M. mazei,* suggesting a potential redundant catalytic activity. The evolutionary connection of casposon-encoded Cas1 proteins and those encoded in the CRISPR-Cas system is further highlighted in *M. mazei* by the interaction of MM_0565 with both the casposon-encoded *cas1-solo* gene and the leader region responsible for the integration of new spacers by the CRISPR-Cas system-encoded Cas1/Cas2 complex, unravelling common regulatory pathways for both genetic elements.

## 5. Conclusions

In conclusion, we showed that the CRISPR-associated protein MM_0565 has global regulatory function but might also influence the CRISPR-Cas system of the methanogenic archaeon *M. mazei* Gö1 on different levels. MM_0565 forms stable dimers under high salt conditions, creating a connection to a higher number of mature crRNAs under these conditions, where it might require a so far uncharacterized allosteric effector. Nevertheless, MM_0565 does not directly regulate the transcription of CRISPR locus I-B-encoded *cas* genes or the crRNA, but we propose that MM_0565 might supports the integration of new spacers into the CRISPR array through direct binding to a conserved sequence in the leader region. The activation of the casposon-encoded standalone *cas1* gene by MM_0565 led to the assumption that MM_0565 might be involved in regulating casposon mobility due to osmotic stress and highlights the evolutionary connection between casposons and CRISPR-Cas systems.

## Figures and Tables

**Table 1:** Differentially transcribed genes identified by RNA-seq of *M. mazei* overproducing MM_0565 in comparison with *M. mazei* containing the respective empty vector pWM321 (vector control). Significantly regulated genes were defined as genes with a log2 fold change ≥ 1.5 and a *p* value adjusted (Padj) ≤ 0.05 using the Deseq2 tool, as described in Section 2. The operon encoding Cas1 solo (*MM_3249*) is highlighted in bold.

	ORF	Gene/Protein	log2 Fold Change	Padj
**Upregulated**				
**Energy metabolism**	MM_1439	Methylcobalamin:coenzyme M methyltransferase MtbA2	1.51	5.69 × 10^−9^
	MM_1438	Monomethylamine corrinoid protein MtmC1	1.55	5.69 × 10^−9^
	MM_1437	Monomethylamine:corrinoid methyltransferase MtmB1	1.86	3.54 × 10^−8^
	MM_1436	Monomethylamine:corrinoid methyltransferase MtmB1 (C-terminal domain)	2.26	2.26 × 10^−13^
	MM_1435	Monomethylamine permease MtmP	2.17	1.05 × 10^−13^
	MM_1434	Monomethylamine permease MtmP (C-terminal domain)	2.27	5.36 × 10^−12^
	MM_1433	Efflux RND transporter permease subunit	2.47	1.72 × 10^−16^
	MM_1694	Dimethylamine:corrinoid methyltransferase MtbB1 (C-terminal domain)	1.54	1.76 × 10^−5^
	MM_1693	Dimethylamine:corrinoid methyltransferase MtbB1	2.00	8.15 × 10^−10^
	MM_0388	Heterodisulfate reductase, subunit HdrC	1.54	2.05 × 10^−9^
	MM_0389	Heterodisulfate reductase, subunit HdrB	1.56	5.50 × 10^−10^
	MM_0753	7-cyano-7-deazaguanine synthase queC	1.56	1.23 × 10^−5^
	MM_1055	Trimethylamine corrionid protein	1.78	0.0005
	MM_2084	CO dehydrogenase/acetyl-CoA synthase, gamma subunit	1.83	7.47 × 10^−12^
	MM_3334	Monomethylamine corrinoid protein MtmC2	1.83	3.26 × 10^−10^
	MM_3335	Monomethylamine:corrinoid methyltransferase MtmB2	2.08	1.44 × 10^−11^
	MM_0465	Cobalamin biosynthesis protein CbiM	1.95	5.94 × 10^−15^
	MM_0337	Tryptophan synthase, beta chain	2.25	2.01 × 10^−20^
	MM_1738	Heme biosynthesis protein	2.25	2.39 × 10^−9^
	MM_1737	Heme biosynthesis protein	3.25	8.66 × 10^−25^
	MM_1733	Metalloproteinase	3.04	1.18 × 10^−8^
	MM_1734	Conserved protein	3.37	1.43 × 10^−14^
	MM_2692	NAD(P)/FAD-dependent oxidoreductase	6.91	2.13 × 10^−66^
**Membrane associated**	MM_1800	queuosine precursor transporter	1.70	3.81 × 10^−5^
	MM_1799	Conserved protein	1.77	8.73 × 10^−9^
	MM_0464	Cobalt transport protein CbiN	2.24	5.21 × 10^−19^
	MM_1362	ABC transporter, periplasmic binding protein	2.43	2.22 × 10^−24^
**Transposons**	MM_0766	Transposase	2.51	3.16 × 10^−22^
	MM_2686	Transposase	2.71	2.25 × 10^−6^
	**MM_3249**	**Cas1-solo**	**3.04**	**1.96 × 10^−24^**
	**MM_3250**	**Hypothetical protein**	**5.09**	**2.33 × 10^−81^**
**Others**	MM_2688	radical SAM protein	4.42	4.20 × 10^−22^
	MM_2693	archaeosine biosynthesis radical SAM protein RaSEA	8.65	1.02 × 10^−12^
**Unknown function**	MM_1613	Conserved protein (DUF111 family protein)	1.53	0.004
	MM_1731	Conserved protein	2.17	2.09 × 10^−8^
	MM_2118	PEF-CTERM sorting domain-containing protein	2.20	3.37 × 10^−17^
	MM_3251	Hypothetical protein	2.28	2.94 × 10^−5^
	MM_1612	Conserved protein	2.40	7.11 × 10^−10^
	MM_2687	Hypothetical protein	4.40	6.85 × 10^−21^
	MM_2691	Hypothetical protein	6.49	6.84 × 10^−56^
**Downregulated**				
**Energy metabolism**	MM_3043	Coenzyme F420 hydrogenase	−1.56	3.00 × 10^−9^
	MM_3042	Coenzyme F420 hydrogenase	−1.68	1.34 × 10^−11^
	MM_2005	Phosphate-binding protein	−1.90	2.67 × 10^−7^
	MM_0977	F420-dependent NADP reductase	−1.98	9.62 × 10^−9^
**Membrane associated**	MM_2576	Ferrous iron transport protein B	−1.58	1.30 × 10^−5^
	MM_1909	Glutathione-regulated potassium-efflux system protein	−5.21	5.30 × 10^−84^
**Transposons**	MM_2590	Transposase	−2.34	1.63 × 10^−8^
	MM_2699	Transposase	−3.16	2.80 × 10^−12^
**Transport**	MM_0077	Metallophosphoesterase	−1.80	1.48 × 10^−10^
**Unknown function**	MM_2575	Hypothetical protein	−1.53	0.006
	MM_2602	Hypothetical protein	−1.59	1.50 × 10^−5^
	MM_0978	Hypothetical protein	−1.64	2.16 × 10^−5^
	MM_1865	Conserved protein	−1.66	8.15 × 10^−10^
	MM_3369	Hypothetical protein	−1.99	0.0004
	MM_1864	Conserved protein	−4.35	5.37 × 10^−77^

## Supplementary Materials

The following are available online at https://www.mdpi.com/2218-273X/10/8/1161/s1, Table S1: Strains and plasmids, Table S2: Primer pairs used for cloning and RT-PCR, Figure S1: Purification of His_6_-MM_0565, Figure S2: Stabilization of His_6_-MM_0565 in buffers with higher salt concentrations, Figure S3: Interaction analysis between purified His_6_-MM_0565 and the promoter region of the *cas I-B* operon (*cas8b*) by microscale thermophoresis (MST), Figure S4: Impact of the overproduction mutant of MM_0565 in *M. mazei* Gö1.
