# Supplementary material for: The CARF Protein MM_0565 Affects Transcription of the Casposon-Encoded cas1-solo Gene in Methanosarcina mazei Gö1
Source: Biomolecules. 2020 Aug 7;10(8):1161. doi: 10.3390/biom10081161 (PMC7465815; doi:10.3390/biom10081161)
Supplement: Supplementary file 1 [file biomolecules-10-01161-s001.pdf]

SUPPLEMENTARY MATERIALS

Table S1. Strains and plasmids.

Strain or plasmid	Genotype or description	Source or reference
**Strain**		
M .mazei Gö1	wild type	DSM No. 3647
*M. mazei**	potential cell wall mutant	[29]
E. coli DH5a	general cloning strain	[30]
E. coli JM109 λpir	general cloning strain	[31]
E. coli BL21-CodonPlus®-RIL	general cloning strain, containing the pRIL plasmid (*ileW*, *leuY*, *proL*)	Stratagene, La Jolla, USA
**Plasmid**		
pCR4-TOPO	general cloning vector	Invitrogen, Darmstadt, GER
pET28a (+)	expression vector	Novagene®, Merck Millipore, Darmstadt, GER
pWM321	general cloning vector	[34]
pRS924	pCR4-TOPO containing *MM_0565*	This study
pRS923	pET28a containing *MM_0565*	This study
pRS893	pDrive containing *pmcrB* + RBS	This study
pRS1031	pDrive containing *pmcrB* + RBS with His-tagged *MM_0565*	This study
pRS1032	pWM321 containing *pmcrB* + RBS with His-tagged *MM_0565*	This study

Table S2. Primer pairs used for cloning and RT-PCR. Attached cleavage sites of restriction enzymes are underlined.

Primer designations	5`→3`
**Mutant construction**	
MM565_NheI_for	GCTAGCATGTCAAAGCTTACACTCAT
MM565_BamHI_rev	GGATCCTTAAATGGAAGCAATTCTTC
**5`RACE analysis**	
5RACE_MM0565_outer	CGGTGTCATAAAGAGCAGTA
5RACE_MM0565_inner	CTTCAAGCGCGTTTTTGAAGG
MM0565_specific	GCTTACACTCATCTCCACCA
**RT-PCR analysis**	
qRT_MM1215_for	TCAAGAGCGAGGGCATGAATG
qRT_MM1215_rev	GCACTACCGAGAACAATAGCC
qRT_MM1621_for	TAGGAGGTTTTCTCGGAAGCG
qRT_MM1621_for	AAGCGTATCTCCATCAAGCCC
qRT_MM2181_for	GCCTCCATGAGAAGAATGCTC
qRT_MM2181_for	CTTCAAGGTCTCCAACTCCTG
qRT_MM3249_for	CAACTACAGAAGAGCCTCAAG
qRT_MM3249_rev	GGAGGAAGCATAGTAGTTAGAAGC
qRT_crRNA_for	AGGGTTTGATAATTTTCCAG
qRT_ crRNA_rev	AAAAGCGGTGTTAAGTCAG
qRT_MM565_for	CCTGTTATTATCTGTGTGACGC
qRT_MM565_rev	ATGACCTGGTTGCCTCTATC
qRT_MM564_for	ATTTTTGATGTGTTGTATGG
qRT_MM564_rev	TTTGTTGAAGAAGGTTTTTG
qRT_MM563_for	GGGGAAGGAAAAAGTCAGTC
qRT_MM563_rev	CATTAATTAGTGCTGCGGTG
qRT_MM562_for	GACTGCTTGGTTTTTAGGG
qRT_MM562_rev	GGAGAAAGCACTTGAATACC
qRT_MM561_for	TGTATGGCACATGACTTTGG
qRT_MM561_rev	ACGGGCATCTTTTACACTTC
qRT_MM560_for	CGGATTAGACGAAGGTTCAA
qRT_MM560_rev	TAAAGCGAGCCAAGGAGTT
qRT_MM559_for	AATGGCAGAAGCAGAAACC
qRT_MM559_rev	AAACCGTCCTGTCAATGTG
qRT_MM558_for	GGGCTTTACAGGGTTCAGA
qRT_MM558_rev	ATTATCTCGTCGCTGACAAG
qRT_MM557_for	AGCAACAGCCTGGTCAAAG
qRT_MM557_rev	TCCGGTAACAGCAATCTACG
**EMSA**	
Leader_IB_for	AGAAAATGCGTAGATTGCTGTTAC
Leader_IIIC_for	GTTTCCAAACCACTAAAAAAAC
KonsLeader_rev	AGGGCAAAAATTTCCGTTATTTG
**Microscale thermophoresis**	
Thermo_MM3250_neu_for	CCGCTCTTACTTATGTACAGAATTGAGTATAACTTTAAG TATAAGTTTGATGTATATTTTGTATATAATT
Thermo_MM3250_neu_rev	AATTATATACAAAATATACATCAAACTTATACTTAAAGT TATACTCAATTCTGTACATAAGTAAGAGCGG
Thermo_Prom_MM0565_1_for	AACTAAATCCCTATATCGTAAAACATTTAACCAAACCCT ACAATACATGACATCTAATGT

Thermo_Prom_MM0565_1_rev	ACATTAGATGTCATGTATTGTAGGGTTTGGTTAAATGTTT TACGATATAGGGATTTAGTT
Thermo_Prom_MM0565_2_for	AGCAAATAGAACCAAACATCAAACATTTAACCAAACCC TACAATACATGACATCTAATGT
Thermo_Prom_MM0565_2_rev	ACATTAGATGTCATGTATTGTAGGGTTTGGTTAAATGTTT GATGTTTGGTTCTATTTGCT
Thermo_Prom_MM0565_3_for	AGCAAATAGAACCAAACATCAACTAAATCCCTATATCG TACAATACATGACATCTAATGT
Thermo_Prom_MM0565_3_rev	ACATTAGATGTCATGTATTGTACGATATAGGGATTTAGT TGATGTTTGGTTCTATTTGCT
Thermo_Prom_MM0565_4_for	AGCAAATAGAACCAAACATCAACTAAATCCCTATATCG TAAAACATTTAACCAAACCCTA
Thermo_Prom_MM0565_4_rev	TAGGGTTTGGTTAAATGTTTTACGATATAGGGATTTAGTT GATGTTTGGTTCTATTTGCT
Thermo_Prom_Cas8b_for	TCTATTTGCTTATTTTTATAATTATTTTTAGAAATGTATAT ATAATATGTTAACTATGAACCTTGATGGT
Thermo_Prom_Cas8b_rev	ACCATCAAGGTTCATAGTTAACATATTATATATACATTT CTAAAAATAATTATAAAAATAAGCAAATAGA
Thermo_Prom_MM3360_for	AAAGATAACTGATGAAATGCGAGAAAATTCGTGAGCA AGATCCACTAAAACAAGGATTGAAGATAAACTG
Thermo_Prom_MM3360_rev	CAGTTTATCTTCAATCCTTGTTTTAGTGGATCTTGCTCAC GAATTTTCTCGCATTTCATCAGTTATCTTT
Thermo_kons_Leader_for	TTTTTCTTGTATTTATAAAGGGTTTGATAATTTTCCAGCA AGAATTTTAGCCCCAAAAAAGGGCTCATTT
Thermo_kons_leader_rev	AAATGAGCCCTTTTTTGGGGCTAAAATTCTTGCTGGAAA ATTATCAAACCCTTTATAAATACAAGAAAAA

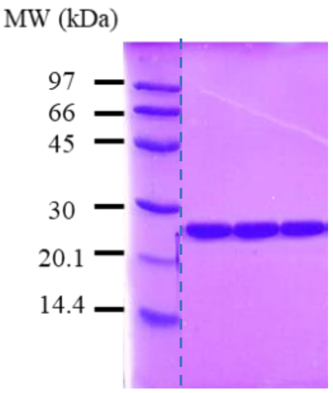

Figure S1. Purification of His6-MM_0565. His6-MM_0565 was heterologously expressed in *E. coli* BL21-CodonPlus®-RIL and purified via Nickel-NTA affinity chromatography. The separation of the samples was performed on a denaturing SDS gel (12%).

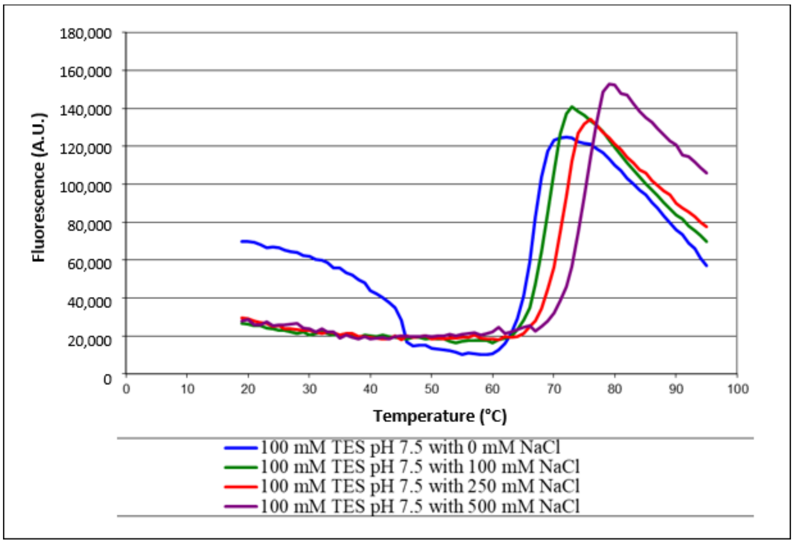

Figure S2: Stabilization of His6-MM_0565 in buffers with higher salt concentrations. Melting curves of His6-MM_0565 in 100 mM TES buffer (pH 7.5) with increasing concentrations of sodium chloride (0, 100, 250 and 500 mM NaCl). Refolding was monitored with fluorescently labeled protein (with SYPRO) in a temperature gradient between 19 and 95 °C (see Material and Methods in the main manuscript).

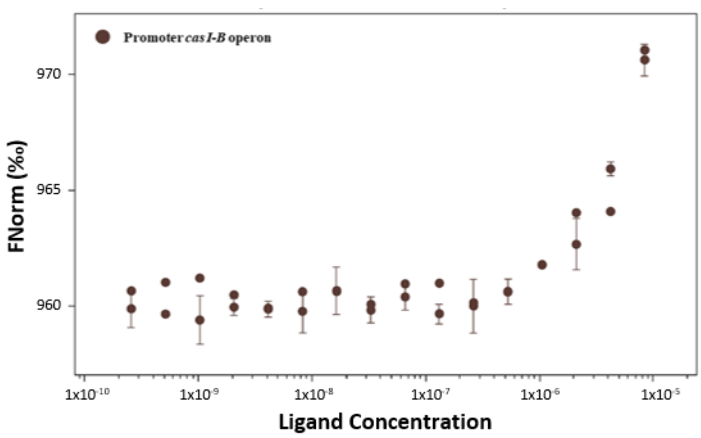

Figure S3: Interaction analysis between purified His$_6$-MM_0565 and the promoter region of the *cas I-B* operon (*cas8b*) by microscale thermophoresis (MST). The interaction between the fluorescent-labeled protein His$_6$-MM_0565 and the promoter region of the *cas I-B* operon (*cas8b*) was measured using the Monolith NT.115 from NanoTemper (Munich, GER) in three independent replicates and evaluated using the corresponding MO Affinity Analysis Software (see the Material and Methods in the main manuscript). K_D values were not calculated due to the fact of no clear binding of MM_0565 to the respective DNA fragments.

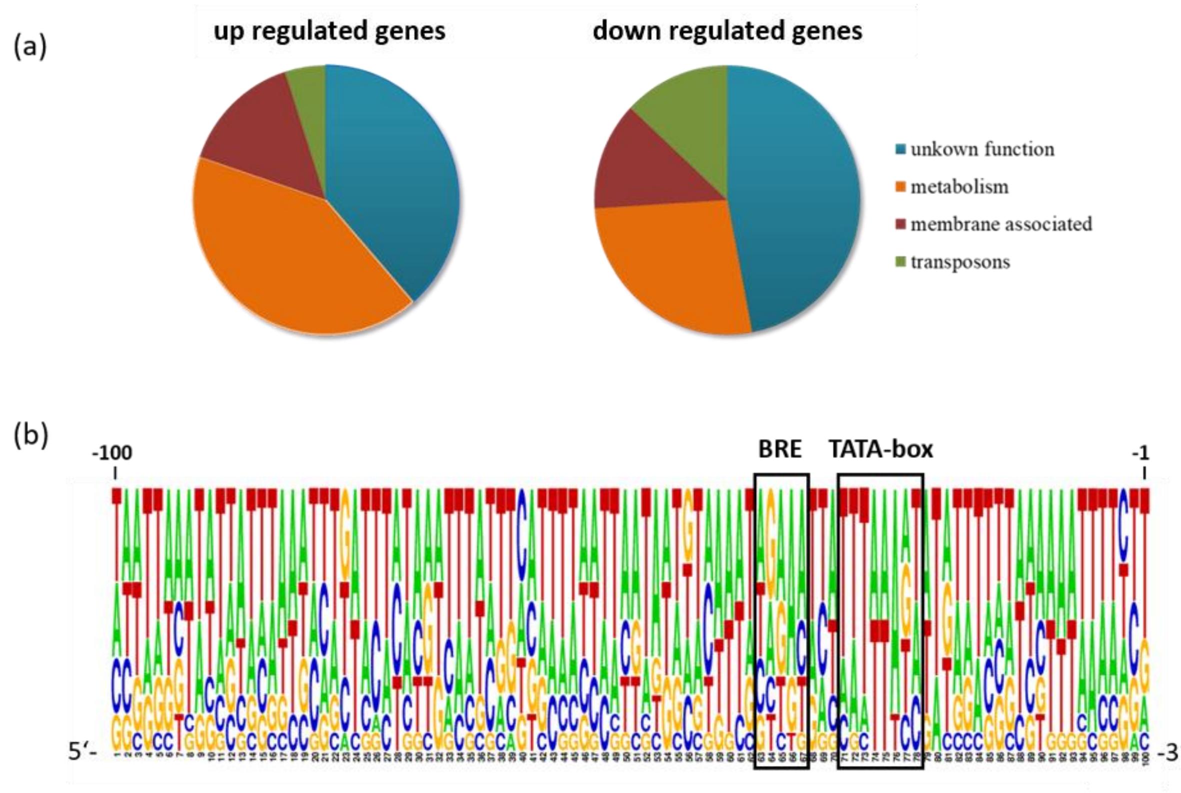

Figure S4: Impact of the overproduction mutant of MM_0565 in *M. mazei* Gö1. (**a**) Classification of the up- (left diagram) and down (right diagram)-regulated genes from the RNAseq data (Table 1, in the main manuscript) according to their function. (**b**) Comparison of promotor regions from genes with an enhanced transcript level in the MM_0565 overproduction mutant. Frequency blot of 100 nucleotides upstream of the transcriptional start sites (TSS) from 14 genes which showed a significantly enhanced transcript level in the *M. mazei* MM_0565 overproduction mutant (see Table 1, in the main manuscript)

using Weblogo [60]. All promotor regions from genes with a distinct TSS in the RNAseq data were used. Putative TATA-boxes and B recognition elements (BRE) are indicated with black boxes.

29. Ehlers, C., et al., *Development of genetic methods and construction of a chromosomal glnK(1) mutant in Methanosarcina mazei strain Gö1.* Mol Genet Genomics, 2005. **273**: p. 290-298.
30. Hanahan, D., *Studies on transformation of Escherichia coli with plasmids.* J Mol Biol, 1983. **166**(4): p. 557-80.
31. Miller, V.L. and J.J. Mekalanos, *A novel suicide vector and its use in construction of insertion mutations: osmoregulation of outer membrane proteins and virulence determinants in Vibrio cholerae requires toxR.* J Bacteriol, 1988. **170**(6): p. 2575-83.
34. Metcalf, W.W., et al., *A genetic system for Archaea of the genus Methanosarcina: liposome-mediated transformation and construction of shuttle vectors.* Proc Natl Acad Sci U S A, 1997. **94**(6): p. 2626-31.
60. Crooks, G.E., et al., *WebLogo: a sequence logo generator.* Genome Res, 2004. **14**(6): p. 1188-90.
